# Complications of Open Elbow Arthrolysis in Post-Traumatic Elbow Stiffness: A Systematic Review

**DOI:** 10.1371/journal.pone.0138547

**Published:** 2015-09-18

**Authors:** Jiangyu Cai, Wei Wang, Hede Yan, Yangbai Sun, Wei Chen, Shuai Chen, Cunyi Fan

**Affiliations:** 1 Department of Orthopedics, Shanghai Jiao Tong University Affiliated Sixth People’s Hospital, 600 Yishan Road, Shanghai, P. R. China, 200233; 2 Division of Plastic and Hand Surgery, Department of Orthopedics, The Second Affiliated Hospital of Wenzhou Medical University, Wenzhou, P. R. China, 325027; Mayo Clinic Minnesota, UNITED STATES

## Abstract

**Objective:**

The objective of this study was to systematically review the literature for a more comprehensive understanding of the complications of open elbow arthrolysis in patients with post-traumatic elbow stiffness and provide a reference for better prevention and treatment of them.

**Methods:**

The PubMed, EMBASE, Cochrane Library, and Google Scholar databases were searched for therapeutic studies with a set of inclusion and exclusion criteria. Data were extracted from selected articles, and a statistical analysis was performed to evaluate related factors and management of the complications.

**Results:**

Twenty-eight articles published between 1989 and 2013, involving 810 patients, were included. Most of the complications included in the selected articles were nerve complications, heterotopic ossification, elbow instability, infection, pin-related complications and repeat elbow contracture. The total complication rate was 24.3% ± 3.0%, and the reoperation rate was 34.0%. Furthermore, the statistical analysis revealed that preoperative range of motion (β = -0.004, P = 0.01) and proportion of female (β = 0.336, P = 0.04) were the independent factors affecting the total complication rate.

**Conclusions:**

Various risk factors are related to each of the complications, and we found that patients with less preoperative ROM and a higher proportion of female gender may point to a higher total complication rate. Therefore, to further improve the overall outcomes of this procedure, more and larger prospective studies should be performed to further elucidate the effects of prophylactic interventions targeting the risk factors, thus improving the methods of prevention and treatment of complications.

## Introduction

Elbow stiffness, a common condition associated with significant morbidity, is generally defined as elbow range of motion (ROM) <30°–130° [1]. It leads to significant limitations and disability related to hand functioning in daily living and work-related activities. The etiology includes atraumatic and traumatic factors [2]. Trauma is known to be the primary cause. The factors associated with elbow stiffness following trauma include prolonged immobilization, soft tissue contracture, intra-articular block, and heterotopic bone formation [3,4].

Both non-operative and operative methods are used to treat elbow stiffness. Generally, surgical release should be considered when non-operative treatment fails to improve the motion after 6 months [5]. Open arthrolysis remains the gold standard for treatment of post-traumatic elbow stiffness. First reported in 1944, the surgical techniques have improved in the past 70 years. Over the years, several reports have described this procedure, with various functional outcomes and complication rates [2,6]. As reported in the literature, there has been a chronological trend in satisfactory outcomes, especially in the recent years, but the occurrence of complications has not decreased with time;this continues to be a thorny issue even for the specialists.

In 2013, Kodde et al. [7] published the first systematic review of operative treatment for post-traumatic elbow stiffness in in journals indexed by MEDLINE. The author focused on comparing the differences between open and arthroscopic arthrolysis and made several suggestions regarding the final outcomes. However, he did not describe the associated complications in detail. Moreover, to our knowledge, no related research was specifically aimed at dealing with the complications associated with open arthrolysis. Therefore, a systematic review of complications related to open arthrolysis is warranted to gain a more comprehensive understanding of these complications and the related factors; this will provide a reference for better prevention and treatment of these complications.

## Materials and Methods

### Search strategy

We searched the PubMed, EMBASE, Cochrane Library, and Google Scholar databases in August 2014 performed an updated search in October, without publication status restrictions, using the following keywords (elbow and [stiff* or contracture or ankylos*] and [releas* or arthrolysis]) to ensure the inclusion of all possible studies. The search was restricted to articles written in English. We also used terms included under Medical Subject Headings (MeSH) in all searches, when possible. In addition, references regarding elbow stiffness were hand-searched for potential studies. All searches were conducted independently by 2 researchers (JYC and WC), and the differences were checked and resolved by discussion.

### Inclusion and exclusion criteria

To be eligible for inclusion, the studies had to: (1) be published clinical trials; (2) meet the diagnostic criteria for post-traumatic elbow stiffness (i.e., elbow ROM <30°–130° after trauma); (3) report operative treatment and outcomes of post-traumatic elbow stiffness in human adults; and (4) reach an average of at least one-year follow-up. Studies were excluded if they: (1) were cadaveric or biomechanical studies, reviews, expert opinions, case reports (number of cases <5), or conference papers that were not published as full reports; (2) reported on stiffness after a burn or central nervous system injury, results of arthroscopic release, or arthroplasty; and (3) were unable to extract the relevant data from the outcomes. We carefully reviewed the full text to exclude studies that partially involved non-posttraumatic stiffness and those that did not mention complications in their follow-up.

### Data extraction

Data were extracted and entered into an Excel database by 2 independent authors (JYC and WC). The data included: author details, year, number of cases, population demographics (age, sex, etc.), mean follow-up, and outcome measures. Disagreements were resolved by involving a third reviewer (WW) in the discussion.

### Outcome measures

The outcome measures were functional outcomes and complications (including types, incidence, and management).

### Statistical analysis

ROM was calculated as mean ± standard deviation (SD). The type-specific complication rates were expressed as percentages with 95% confidence intervals (CIs). We also divided the patients into two groups based on whether they had been treated with hinged external fixation. A Chi-square test or Fisher exact test was applied for comparison of the complication rates of the two groups. Stepwise multiple linear regression analysis was conducted to determine the independent factors (age, sex (female %), time from injury, duration of follow-up, preoperative ROM, postoperative ROM) affecting the total complication rate. The significance level was set at P < 0.05. The statistical analyses were performed using SPSS 19.0 statistical software (SPSS Inc., IL, USA).

## Results

### Study selection

**Fig 1:**
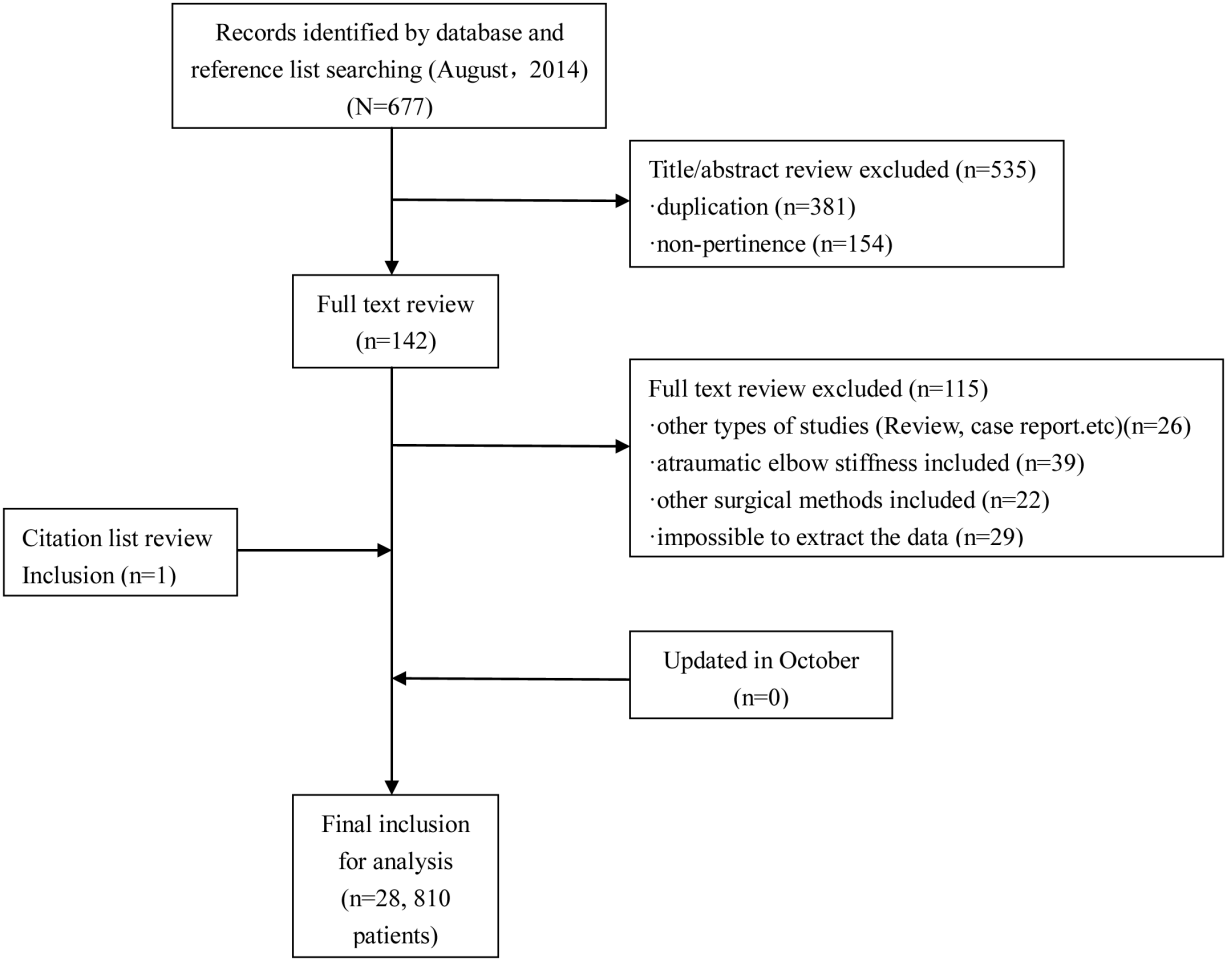
Flow diagram of study selection.

A total of 677 studies were identified in the initial search. After a careful review of the lists, full texts were retrieved for 142 articles. We subsequently excluded 115 articles that did not meet the inclusion criteria. A search of the reference lists of selected articles identified 1 more relevant article; our search was updated in October 2014 with no more relevant articles, leaving a total of 28 articles, involving 810 patients (814 elbows) [5,8–34], for the final inclusion (Fig 1).

### Characteristics of included studies and functional outcomes

The main characteristics of the studies included in the systematic review are summarized in Table 1. Since no randomized controlled trials were found, data could not be analyzed by meta-analysis. Of the included studies, 4 were retrospective cohort studies by design (level 3), and the rest were case series (level 4), as assessed by the Oxford Centre for Evidence-Based Medicine 2011 Levels of Evidence [35]. The number of patients in the studies ranged from 7 to 81. These articles were published between 1989 and 2013. We also extracted the data on ROM as the functional outcomes. The mean preoperative ROM in these studies was 51° ± 15°, and the mean postoperative ROM was 107° ± 9°. The patients had shown an average ROM increase of 56° ± 14°.

**Table 1:** Characteristics of selected studies and functional outcomes.

Author (Year)	Level of evidence	No. of Patients	Mean Age (years)	Mean Interval from Injury (months)	Sex, female %	Mean FU (months)	Mean Pre-ROM (°)	Mean Post-ROM (°)	Mean Gain in ROM (°)
Ruan (2013) [8]	4	15	38	20	40.0	31	0	116	116
Ouyang (2013) [9]	4	11	42	22	41.7	29	41	114	73
Koh (2013) [10]	4	24	39	26	54.2	60	60	105	45
Koh (2013) [11]	4	77	38	19	43.4	42	45	112	67
Wang (2012) [12]	4	25	29	10	32.0	16	33	97	64
Malone (2012) [13]	3	Without UCL res 18	35	45	11.0	25	55	115	60
		With UCL res & rec 6	42	10	33.0	20	28	100	72
Higgs (2012) [5]	4	81	40	36	33.3	15	69	109	40
Liu (2011) [14]	4	12	34	9	36.4	15	35	115	80
Ayadi (2011) [15]	4	22	31	16	20.0	56	51	95	44
Park (2010) [16]	4	42	37	10	52.4	39	55	115	60
Lindenhovius (2010) [17]	4	22	44	21	31.8	23	51	106	55
Nobuta (2008) [18]	4	27	42	14	33.3	18	53	95	42
Gundlach (2008) [19]	4	21	40	NR	52.4	24	69	104	35
Sharma (2007) [20]	4	25	34	13	24.0	94	55	105	50
Lindenhovius (2007) [21]	3	With HO 16	43	10	50.0	26	59	116	57
		Without HO 21	51	9	38.1	30	52	98	46
Tan (2006) [22]	4	52	35	14	38.5	19	57	116	59
Ring (2006) [23]	4	46	45	16	50.0	48	45	103	58
Darlis (2006) [24]	4	12	17	21	16.7	19	62	116	54
Cikes (2006) [25]	4	18	36	12	72.2	16	82	122	40
Ring (2005) [26]	3	With ext fix 23	36	15*	34.8	39*	21	107	86
		Without ext fix 19	43		31.6		24	103	79
Park (2004) [27]	3	With HO 18	33*	15*	33.3*	23*	50	110	60
		Without HO 9					47	86	39
Marti (2002) [28]	4	46	31	21	50.0	120	45	99	54
Bae (2001) [29]	4	12	16	35	7.7	30	53	107	54
Cohen (1998) [30]	4	22	35	70	45.5	29	74	129	55
Boerboom (1993) [31]	4	14	36	22	41.7	62	73	112	39
Amillo (1992) [32]	4	34	31	19	36.4	62	45	92	47
Husband (1990) [33]	4	7	32	14	14.3	38	71	117	46
Weizenbluth (1989) [34]	4	13	29	60	38.5	60	34	85	51

FU, follow-up; Pre-ROM, preoperative range of motion; Post-ROM, postoperative range of motion.

UCL, ulnar collateral ligament; res, resection; rec, reconstruction; HO, heterotopic ossifications; ext fix, external fixation; NR, not recorded

* These studies only shown data in total instead of respective data of each subgroup.

### Complications

Among the 810 patients, the total number of complications after open elbow arthrolysis was 197 (24.3% ± 3.0%), which included nerve complications, heterotopic ossification (HO), infection, and elbow instability and others. 67 patients (34.3%) needed reoperations because of the persistent symptoms after the index release. The complications are recorded from each of the selected studies in Table 2 and summarized in Table 3. Details of the complications are shown below.

**Table 2:** Breakdown of complications reported in each of the selected studies.

Author (Year)	Reported complications rate (No.)	Nerve complications	Heterotopic ossification	Elbow instability	Infection*	Pin-related complications^#^	Others
Ruan (2013) [8]	40% (6)	0	3 Rd	1	0	2 Pinf	0
Ouyang (2013) [9]	18.2% (2)	1R	0	0	0	1 Pinf	0
Koh (2013) [10]	20.8% (5)	0	1 Cl	0	0	0	4 refracture
Koh (2013) [11]	35.1% (27)	9U	16 (9 Rd,7 Cl)	1	1	0	0
Wang (2012) [12]	8.0% (2)	1U	0	0	0	1 Pfra	0
Malone (2012) [13]	12.5% (3)	1U	0	0	0	0	2 hematoma
Higgs (2012) [5]	1.2% (1)	0	0	0	1	0	0
Liu (2011) [14]	25.0% (3)	2U	0	1	0	0	0
Ayadi (2011) [15]	18.2% (4)	4(1R,2U,1M)	0	0	0	0	0
Park (2010) [16]	59.5% (25)	9U	12 (10 Rd,2 Cl)	3	1	0	0
Lindenhovius (2010) [17]	31.8% (7)	4U	3 Cl	0	0	0	0
Nobuta (2008) [18]	18.5% (5)	4U	1 Cl	0	0	0	0
Gundlach (2008) [19]	19.0% (4)	0	2 Rd	0	1	0	1 REC
Sharma (2007) [20]	20.0% (5)	4U	0	0	0	0	1 triceps avulsion
Lindenhovius (2007) [21]	With HO 37.5% (6)	3U	2 Rd	0	0	0	1 REC
	Without HO 33.3% (7)	3U	1 Cl	0	0	0	3 REC
Tan (2006) [22]	26.9% (14)	4 (3U,1RSD)	0	2	3	0	5 REC
Ring (2006) [23]	28.3% (13)	4U	0	0	0	0	9 REC
Darlis (2006) [24]	16.7% (2)	0	1 Rd	0	1	0	0
Cikes (2006) [25]	16.7% (3)	0	0	0	1	0	1 hematoma, 1 wound dehiscence
Ring (2005) [26]	With ext fix 56.5% (13)	0	0	0	0	6 Pinf, 2 Pbrk, 1 Pfra, 2 PU	2 REC
	Without ext fix 26.3% (5)	0	0	0	0	0	5 REC
Park (2004) [27]	18.5% (5)	1U	3 (1 Cl,2 Rd)	0	1	0	0
Marti (2002) [28]	28.3% (13)	7U	0	0	2	0	4 REC
Bae (2001) [29]	16.7% (2)	1U	0	0	0	0	1 incomplete wound healing with a sinus
Cohen (1998) [30]	27.3% (6)	4 (3U,1M)	0	0	0	0	2 synovitis
Boerboom (1993) [31]	7.1% (1)	0	0	0	0	0	1 REC and dislocating radial head
Amillo (1992) [32]	5.9% (2)	1U	0	0	1	0	0
Husband (1990) [33]	14.3% (1)	1U	0	0	0	0	0
Weizenbluth (1989) [34]	38.5% (5)	2U	0	0	0	0	2 REC and 1 intraarticular bleeding

R, radial nerve complications; U, ulnar nerve complications; M, median nerve complications; RSD, Reflex Sympathetic Dystrophy.

Rd, radiographic new-onset or recurrence recurrence; Cl, clinical new-onset or recurrence; REC, repeat elbow contracture.

* excluding pin track infection

^#^ including Pinf, Pbrk, Pfra and PU. Pinf, pin track infection; Pbrk, pin breakage; Pfra, pin-site fracture; PU, pin-related ulnar nerve irritation.

**Table 3:** Summary of the reported complications.

	Reported complications (No.)	Complication rate	Reoperation rate (No.)	Method of Reoperation
Nerve complications	70	8.6% ± 1.9%	18.6% (13)	13 ulnar nerve decompression
Heterotopic ossification	45	5.6% ± 1.6%	28.9% (13)	12 elbow release and HO excision
				1 interposition arthroplasty
Elbow instability	8	1.0% ± 0.7%	25.0% (2)	2 external fixation placement
Infection	13	1.6% ± 0.9%	53.8% (7)	7 irrigation and debridement
Pin-related complications	15	17.4%± 8.0%	13.3% (2)	1 (osteomyelitis) debridement
				1 (fracture) ORIF
Others*	46	5.7% ± 1.6%	63.0% (29)	4 (refracture) ORIF
				22 (REC) open arthrolysis
Total	197	24.3% ± 3.0%	34.0% (67)	—

*including repeat elbow contracture, refracture, hematoma, synovitis, tricep avulsion, incomplete wound healing with a sinus, wound dehiscence and intrarticular bleeding.

ORIF, open reduction and internal fixation

#### Nerve complications

Nerve complications, defined as new-onset or exacerbation of nerve symptoms postoperatively, occurred in 70 patients (8.6% ± 1.9%) and consisted of ulnar, radial, and median nerve symptoms and reflex sympathetic dystrophy. We found that nerve complications involving the ulnar nerve, also known as delayed-onset ulnar neuropathy (DOUN), were the most common (66 patients); thereafter, 13 of 70 patients (18.6%) needed a subsequent surgery to decompress the ulnar nerve with or without transposition.

#### HO

HO occurred in 45 patients (5.6% ± 1.6%) and consisted of radiographic and clinical new-onset or recurrence. Patients who failed to regain a functional arc of motion due to HO postoperatively are considered to have clinical new-onset or recurrence. 13 of 45 patients (28.9%) underwent a second surgery for HO, and all of them had clinical recurrence of HO.

#### Elbow instability

Elbow instability occurred in 8 patients (1.0% ± 0.7%), which included valgus and varus instability, as well as elbow subluxation. Hinged external fixators were placed at 3 and 7 weeks post-release in 2 of 8 patients (25.0%), and the remaining patients with mild instability that did not affect daily function did not require surgical treatment.

#### Infection

Infection occurred in 13 patients (1.6% ± 0.9%), which included superficial infection and deep infection (pin track infection was excluded). Seven of the 13 patients (53.8%) underwent a reoperation as irrigation and debridement. As a result, the infection was under control in 3 and the outcomes of the remaining 4 patients were not reported in the studies.

#### Pin-related complications

Among the 86 patients with routine hinged external fixation, pin-related complications occurred in 15 patients (17.4% ± 8.0%), which included 9 patients with pin track infection, 2 with pin breakage, 2 with ulnar nerve irritation, and 2 with pin-site fracture. Two patients (13.3%) needed a second surgery: 1 patient with pin track osteomyelitis underwent debridement and 1 with a pin-site fracture was treated with open reduction and internal fixation.

#### Other complications

The other complications included 33 repeat elbow contractures for unknown or unreported reasons (excluding elbow contracture associated with HO mentioned in the studies), 4 re-fractures (intercondylar region of the distal humerus), 3 hematomas, 2 cases of synovitis, 1 tricep avulsion, 1 incomplete wound healing, 1 wound dehiscence and 1 intrarticular bleeding. Twenty-nine patients (63.0%) had subsequent surgeries to deal with the complications: 4 patients with a re-fracture and 25 patients with a repeat elbow contracture. The other 8 with a repeat elbow contracture refused a second arthrolysis.

#### Correlation of complications

There was a significant difference in the reoperation rate between the patients treated with and without hinged external fixation (P = 0.04). However, difference of total complication rate between the two groups was not significant (P = 0.18) (Table 4). Results from the multiple linear regression analysis revealed that preoperative range of motion (β = -0.004, P = 0.01) and proportion of female (β = 0.336, P = 0.04) were the independent factors affecting the total complication rate. However, no significant association was found between the complication rates and other clinical variables, including age, time from injury, duration of follow-up and postoperative ROM.

**Table 4:** Summary of patients treated with and without hinged external fixation.

Hinged external fixation	No. of studies	Gain ROM (°)	Pin-related complication rate	Total complication rate	P	Reoperation rate	P
Yes (n = 86)	5	81	17.4% ± 8.0%	30.2%	0.18	15.4%	0.04
No (n = 724)	23	53	—	23.6%		36.3%	

## Discussion

In this systematic review, we have examined the complications following open elbow arthrolysis for post-traumatic elbow stiffness. Twenty-eight studies involving 810 patients were identified for the final inclusion.

### 1. Nerve complications

Nerve complications, especially DOUN, were most common in the examined studies. The associated clinical symptoms are attributable to several factors. First, the procedure can cause iatrogenic injury of the nerve when the dissection is not meticulous or complete [24,30]. Second, the increase in length and intra-neural pressure of the ulnar nerve with increasing ROM postoperatively may contribute to neuropathy [17,30]. In addition, the normal fibro-osseous tunnel in which the ulnar nerve lies is more susceptible to swell, thicken, and scar after surgical intervention [36]. Since the ulnar nerve is very susceptible to pathologic changes around the elbow and ulnar neuropathy is a strong predictor of improvement in health status after open elbow arthrolysis [17], application of an appropriate ulnar nerve intervention is important during the arthrolysis. As mentioned in the selected studies, the indications for ulnar nerve release included preoperative ulnar nerve symptoms [11,13,16,17,23,24,27,30], surgery using the medial approach [5,21,28,29], nerve entrapment at the time of the surgery [16,17,27], severe flexion contracture [11,22], large osteophytes or HO on the medial aspect [11,22], an inappropriate ulnar nerve bed [5,16], and positive provocative tests for impingement of the ulnar nerve [30]. Blonna et al. [37] revealed that the significant risk factors for DOUN after arthroscopic elbow release included a diagnosis for HO, preoperative neurological symptoms and preoperative arc of motion through a case-control study of 565 cases. However, some studies also reported that patients without the above-mentioned indications had DOUN. Ring et al. [23] reported 2 patients with preoperative elbow flexion ≥100° (100° and 120° respectively), who developed DOUN and underwent subsequent surgery. Routine ulnar nerve intervention may be a reasonable approach during the index elbow release to prevent DOUN. Three studies [8,9,14] reported decompression in all patients, and the results indicated that none of them had DOUN at the last follow-up. A highly related study from our institution reviewed 94 patients and summarized our experience in dealing with the ulnar nerve in open elbow arthrolysis [38]. The study suggested that routine decompression and subcutaneous anterior transposition were indispensable in the prevention of DOUN in the treatment of the stiff elbow. Moreover, Blonna et al. [39] also suggested routine decompression of the ulnar nerve during arthroscopic release. Moreover, they also noted that the decompression length of the ulnar nerve was important to the efficacy of prophylactic intervention, and a mini-decompression (4 to 6 cm) was less effective compared with a decompression of 7 cm or longer. This finding suggested that ulnar nerve decompression to sufficient length was significant for reducing the incidence of DOUN.

### 2. HO

HO is a major complication, and its specific mechanism is still unclear. Since many studies did not report the results of the postoperative radiographic evaluation, the radiographic new-onset or recurrence of HO might have been neglected by the authors, and the actual incidence was much higher than expected. HO of the elbow can develop after direct trauma (fracture, dislocation, or both), central nervous system injury, burn injury, and among those with genetic diseases. Furthermore, surgical dissection, which may lead to soft-tissue insult, is also related to the development of HO [40]. Clinical new-onset or recurrence of HO is one of the factors responsible for the failure to restore ROM and a possible factor predicting poor results in arthrolysis for stiff elbows caused by HO [11]. The prophylactic interventions for preventing recurrence of HO that were suggested in the selected studies included radiotherapy [11,16,17,24,27], non-steroidal anti-inflammatory drugs (NSAIDs) [5,8,9,14,18,22,25,28,30,31] or both [12,15,21,29]. In addition, one study mentioned that a sharp epiperiosteal resection around the ossification was essential to prevent recurrence of HO [18]. However, we could not identify the indications and appropriate group with specific data of patients for prophylactic interventions after open elbow arthrolysis due to the low evidence of the studies. According to the findings from elbow, acetabular and hip surgeries, a high body mass index (BMI), longer injury time and preoperative ROM were the risk factors for development of HO [41–45]. These findings may be helpful to determine the use of prophylactic interventions for specific patients of elbow stiffness. Although most patients who had undergone these interventions were reported to have gained satisfactory ROM with a low rate of recurrence in the selected studies there were few comparative trials to better evaluate the efficacy of these interventions for preventing recurrence of HO after open elbow arthrolysis. Only one study conducted by Koh et al.[11] reported the rate of HO recurrence was 12.5% in patients who received postoperative radiation prophylaxis and 34.5% in patients who did not receive radiation prophylaxis respectively, which showed a significant difference (P = 0.02). More comparative trials should be carried out to support the use of radiotherapy and NSAIDs. Besides the interventions mentioned above, Viola et al. reported that the rate of HO recurrence of the elbow could be reduced by meticulous surgical techniques (including the best-designed surgical approach to sharply resect HO, a less invasive soft tissue intervention and adequate hemostasis) and wound drainage [46].

### 3. Elbow instability

Elbow instability is a common complication after complete arthrolysis for severe elbow stiffness. With a careful stress test for stability during the surgery as well as the development of the surgical techniques, instability could be recognized and avoided. The main causes of postoperative instability are the initial injuries and iatrogenic violation of the collateral ligaments, which play a vital role in maintaining elbow stability [23]. Generally, during the release, the anterior band of the medial collateral ligament and the lateral collateral ligament should be preserved as the primary stabilizers of the elbow, according to the early biomechanical [47–49] and clinical studies [5,10,13,14,16,22,24,27,28,30]. As found in the selected articles, mild postoperative instability is often resolved in approximately 2 months, which is probably due to the dynamic contribution of the periarticular musculature [50,51]. In some severe cases, for instance, if the collateral ligaments were severely contracted or encased in HO,the ligaments had to be resected and then repaired, reattached, or reconstructed for increase in ROM as well as stability. The pullout suture or suture anchor repair technique was used in 3 studies to reconstruct the proximal origin of collateral ligaments as well as reattach the flexor-pronator and extensor tendons to the epicondyle and supracondylar ridge [11,24,27]. However, the authors did not focus much on evaluating the efficacy of this technique. One study from our institution reviewed 46 cases of severely stiff elbows undergoing open release and ligament reconstruction with suture anchor repair technique [52]. The technique proved to fulfill the biomechanical requirements and achieve satisfactory outcomes for patients whose ligaments could not be repaired directly during operation.

In order to achieve greater stability after extensive release, a hinged external fixator was applied during the surgery in 5 studies and was maintained in place for 4–8 weeks [8,9,12,14,23]. The advantages of using a hinged external fixator mentioned in the studies were: (1) to provide stability; (2) to meet the needs of the early rehabilitation; (3) to stretch the contracted soft tissues and distract the articular surfaces; (4) to help maintain the motion obtained during operation. However, it was associated with concomitant pin-related complications including pin track infection, pin breakage, and pin-site fracture. As was recorded in our study, the rate of pin-related complications was relatively high (17.4% ± 8.0%). However, according to Table 4, the reoperation rate was significantly higher in patients treated with a hinge (36.3% vs 15.4%, P = 0.04) and the total complication rate was statistically insignificant between the two groups (23.6% vs 30.2%, P = 0.18). It seemed that the application of hinged external fixator could protect patients from other complications as well as reoperations and might not add extra risk of complications to the patients. Furthermore, the mean gain in ROM was higher in the group with hinged external fixation than the other group (81° vs 53°). Based on the results of statistical analysis, we believe that the advantages of using a hinged external fixator outweigh the disadvantages for severe elbow stiffness, as long as with correct application of the device intraoperatively and careful local care of the pin track postoperatively.

### 4. Infection

The occurrence of infection as a complication cannot be underestimated in any joint surgery, including open elbow arthrolysis. It can spiral out of control, and subsequent disability is inevitable. As recorded above, 7 of 13 patients underwent a second surgery and achieving satisfactory outcomes was difficult. In the selected studies, the treatment for infection consisted of intravenous or oral antibiotics as well as irrigation and debridement. Regrettably, the risk factors and more detailed treatments were not described. One study from our institution showed that the severity of elbow stiffness with less active ROM, longer operative time, diabetes and increased previous operation times seemed to be potential risk factors for infection after open elbow arthrolysis [53]. Moreover, the study introduced a prophylactic method of intrawound application of vancomycin powder for prevention of infection during open elbow arthrolysis. This method was reported to significantly decrease postoperative infection rate compared with the use of standard intravenous antibiotics after open elbow arthrolysis (6.5% vs 0%, P = 0.00). In addition, Miller et al. [54] described a technique for the delivery of regional antibiotic prophylaxis for elbow surgeries including elbow arthrolysis. The regional antibiotic prophylaxis achieved higher tissue antibiotic concentrations compared with those achieved with standard systemic antibiotic, which might help reduce the risk of infection in elbow arthrolysis.

### 5. Other complications

We reviewed all the studies [19,21–23,26,28,31,34] on repeat elbow contractures due to unknown or unreported factors. The factors associated with repeat elbow contractures were recurrence of soft tissue contracture and the new onset or recurrence of HO, which are similar to the initial causes for post-traumatic elbow stiffness. The development of HO has been discussed previously. According to the studies, recurrence of soft tissue contracture was intimately linked with the surgical technique and postoperative rehabilitation regimes. Two study [23,31] mentioned capsulectomies in the treatment of the repeat elbow contracture. We supposed that excision of the capsule of the contracted elbow during the index surgery was not sufficient in some cases. Furthermore, early and appropriate postoperative rehabilitation played a key role in achieving mobility according to some studies [5,8,9,14,28,30,34]. Weizenbluth et al. [34] reported that the lack of cooperation with postoperative rehabilitation would predict occurrence of repeat elbow contracture. In addition, the occurrence of a re-fracture was reported primarily by Kohn et al.’s study [10] in which arthrolysis was performed along with implant removal. The author indicated that the potential risk of fracture should be taken into account when these 2 procedures were performed together. This may also be an important point of consideration for other surgeons. The other complications such as hematoma, synovitis, tricep avulsion, incomplete wound healing, wound dehiscence and intrarticular bleeding mentioned above also should be prevented and treated with caution by the surgeons. In addition, arthrosis is a potential but crucial complication that may occur and have an adverse influence on the long-term status. However, it was difficult to evaluate the occurrence of arthrosis because of the few details reported and the relatively short follow-up duration.

Based on the results of the multiple linear regression analysis, preoperative ROM (β = -0.004, P = 0.01) and proportion of female (β = 0.336, P = 0.04) were the independent factors affecting the total complication rate. Less preoperative ROM, associated with higher severity level of elbow stiffness, was one of the risk factors for many complications including DOUN, HO, elbow instability and infection discussed above. Therefore, it would significantly predict a higher total complication rate. In this regard, surgeons should pay more attention to the patients with less preoperative ROM, and consider the use of prophylactic treatment to reduce complication rate. Meanwhile, the results also showed a higher proportion of female was associated with a higher total complication rate. We assume the different anatomy of the elbow between male and female may primarily account for this finding. This is another point of interest for future research.

Although most of these studies did not report the results of subsequent surgeries, to our knowledge, surgeries performed after the index surgery usually do not have satisfactory outcomes. Therefore, more emphasis should be placed on the persistent complications, especially those with a relatively high operation rate, including repeat elbow contracture (75.8%), infection (53.8%) and HO (28.9%). Complication-related reoperation will lead to decreased confidence among patients as well as the surgeons. More importantly, the persistent complications would increase the economic and psychological burden on the patients. This is why use of several prophylactic interventions for preventing complications, especially the persistent ones, is important. In our view, prophylactic interventions tend to exert an increasingly important effect on the safety and outcome of open elbow arthrolysis.

This systematic review has some limitations that should be taken into account. First, since the studies identified were level 3 or level 4 on the level of evidence, the general strength of the evidence from these studies was not of the highest quality. Second, as in other meta-analyses or systematic reviews that study specific outcomes, there exists the inherent possibility of publication bias; a lower complication rate or better ROM, for example, may be more likely to be published. Third, though the studies were selected strictly on the basis of inclusion and exclusion criteria, there was a lack of homogeneity in the severity of the stiff elbow, surgical techniques, rehabilitation, and duration of follow-up among the studies and it was difficult to perform a more specific statistical analysis between patients and each of the complication. We must declare, however, that the characteristics of complications were arrived at based on the findings of 28 studies. Moreover, further discussion of the risk factors and prevention and treatment strategy in this study will provide a reference to reduce the complication rate and improve the outcomes of open elbow arthrolysis.

## Conclusions

In summary, the complications associated with open elbow arthrolysis mainly include nerve complications, HO, elbow instability, infection, pin-related complications and repeat elbow contracture. Various risk factors are related to each of the complication. According to the literature review, a diagnosis of HO, preoperative nerve symptoms and preoperative ROM are risk factors for nerve complications; a high BMI, longer injury time and preoperative ROM are the risk factors for HO; higher severity level of stiffness is a risk factor for elbow instability; severity of stiffness with less ROM, longer operative time, diabetes and increased previous operation times are risk factors for infection. Besides, the statistical analysis reveals that patients with less preoperative ROM and a higher proportion of female gender may point to a high total complication rate. Moreover, the persistent complications with a significant reoperation rate and an unsatisfactory outcome are difficult to handle. Therefore, to further improve the overall outcomes of this procedure, more and larger prospective studies should be performed to further elucidate the effects of prophylactic interventions and to determine which combination of risk factors can be effectively ameliorated by prophylactic interventions like ulnar nerve decompression for nerve symptoms, radiotherapy and NSAIDs for HO, application of suture anchor repair technique and external fixation for instability and the regional antibiotic prophylaxis for infection, thus improving the methods of prevention and treatment of complications, especially the persistent ones.

## Supporting Information

S1 PRISMA Checklist PRISMA checklist. (DOC)
